# Explainable Artificial Intelligence Model for Stroke Prediction Using EEG Signal

**DOI:** 10.3390/s22249859

**Published:** 2022-12-15

**Authors:** Mohammed Saidul Islam, Iqram Hussain, Md Mezbaur Rahman, Se Jin Park, Md Azam Hossain

**Affiliations:** 1 Network and Data Analysis Group, Department of Computer Science and Engineering, Islamic University and Technology (IUT), Gazipur 1704, Bangladesh; 2 Department of Biomedical Engineering, Medical Research Center, College of Medicine, Seoul National University, Seoul 03080, Republic of Korea; 3 Data Mind Ltd., Dhaka 1230, Bangladesh; 4 AI-Based Healthcare Research Group, Sewon Intelligence Ltd., Seoul 04512, Republic of Korea

**Keywords:** explainable AI, electroencephalography, stroke, machine-learning, LIME, Eli5

## Abstract

State-of-the-art healthcare technologies are incorporating advanced Artificial Intelligence (AI) models, allowing for rapid and easy disease diagnosis. However, most AI models are considered “black boxes,” because there is no explanation for the decisions made by these models. Users may find it challenging to comprehend and interpret the results. Explainable AI (XAI) can explain the machine learning (ML) outputs and contribution of features in disease prediction models. Electroencephalography (EEG) is a potential predictive tool for understanding cortical impairment caused by an ischemic stroke and can be utilized for acute stroke prediction, neurologic prognosis, and post-stroke treatment. This study aims to utilize ML models to classify the ischemic stroke group and the healthy control group for acute stroke prediction in active states. Moreover, XAI tools (Eli5 and LIME) were utilized to explain the behavior of the model and determine the significant features that contribute to stroke prediction models. In this work, we studied 48 patients admitted to a hospital with acute ischemic stroke and 75 healthy adults who had no history of identified other neurological illnesses. EEG was obtained within three months following the onset of ischemic stroke symptoms using frontal, central, temporal, and occipital cortical electrodes (Fz, C1, T7, Oz). EEG data were collected in an active state (walking, working, and reading tasks). In the results of the ML approach, the Adaptive Gradient Boosting models showed around 80% accuracy for the classification of the control group and the stroke group. Eli5 and LIME were utilized to explain the behavior of the stroke prediction model and interpret the model locally around the prediction. The Eli5 and LIME interpretable models emphasized the spectral delta and theta features as local contributors to stroke prediction. From the findings of this explainable AI research, it is expected that the stroke-prediction XAI model will help with post-stroke treatment and recovery, as well as help healthcare professionals, make their diagnostic decisions more explainable.

## 1. Introduction

Acute ischemic stroke and intracerebral hemorrhage are two of the leading causes of neurological disease in the elderly, exposing millions of individuals to neurological abnormalities and physical impairments [1,2]. An ischemic injury impairs the functional network architecture of cortical regions, resulting in decreased motor and cognitive performance [3]. Stroke-related neurological damage adds to disability, poor functional recovery, and reduced quality of life. Furthermore, cognitive deficiency can limit the effectiveness of post-stroke therapy and significantly raise the risk of psychiatric illnesses such as depression and anxiety. The economic impact of post-stroke care is much higher in patients with physiological impairment than in those without. Exact assessment of factors that predict cognitive and functional results is required for making medical decisions, creating achievable rehabilitation objectives and programs, and directing patients appropriately.

Cortical activity plays a vital role in terms of identifying stroke patients [4]. The neuro-electrical activity of the stroke-impaired cortical lobes destabilizes the whole neural system [5]. Functional motor and cognitive impairments are common and long-term consequences of a stroke. They play a big role in the development of physical disability, the slowing of physical rehabilitation, and the worsening of quality of life after a stroke. Classic psychological and neurological tests cannot be performed right after a stroke because of medical problems (such as different levels of arousal, pain, uncertainty, and fatigue) and activity impairments (such as motor, linguistic, and sensory deficits) that make it hard for the patient to face physical tests [6].

Electroencephalography (EEG) is a non-invasive imaging technology that has limited spatial resolution but excellent temporal resolution. Irregularities in brain rhythms induced by a stroke are easily identifiable by an EEG wave, making it a useful and alternate diagnostic tool for cognitive assessments [7]. In everyday life, the physiological signal can serve as a useful instrument for real-time physiological monitoring and early prognosis [4,8,9,10,11]. Several EEG studies have been published to investigate the relationship between EEG markers and neurologic prognosis after ischemic stroke in medical and healthcare settings [3,6,12,13,14,15,16,17,18].

With the advancement of the internet of things (IoT), wearable devices, digital twins, cyber-physical systems, big data, and Healthcare 4.0 in medicine, a real-time biosignal-based patient monitoring system draws much attention [19]. Big-ECG, a cyber-physical cardiac monitoring system, was proposed for stroke prognosis and post-stroke patient monitoring [20]. HealthSOS, a real-time health monitoring system for stroke prognostics was proposed which consists of an eye-mask embedded portable EEG device, data analytics, and medical ontology-based health advisor service [21]. A healthcare “digital twin” framework has been suggested for stroke diagnostics using a wearable EEG and investigating neurological parameters in various mental states [22]. EMG and pressure insole-based gait monitoring systems were investigated consisting of a portable EMG device, cloud-based data processing, data analytics, and a health advisor service for stroke patients [23,24].

The recent successful adoption of artificial intelligence (AI) in healthcare and medical facilities has been mostly driven by machine learning (ML) and deep learning (DL). ML and DL have revolutionized how we approach real-world tasks that were traditionally performed by humans. These models are powerful tools that can be used for a variety of purposes, including classification, clustering, recommendation, ranking, forecasting, and so on. However, these techniques are complex and very difficult to interpret due to their diversity and nature. Most of the ML models act as “black box” models, where we feed some input to the model, and it gives some output at the end. The lack of transparency in ML models makes it harder for users to accept them and limits their use in sensitive fields like healthcare, finance, and law, where users need explanations to understand and interpret the results. In this regard, Explainable Artificial Intelligence (XAI) is presented as a technical solution to find out what the ML models learn during training and how decisions for specific or new instances are made during the prediction phase.

Our study attempts to develop an effective stroke prediction ML model that utilizes EEG data and provides visual interpretability of the findings in ML models. Local and global explanations are the two primary mechanisms by which XAI approaches provide model explanations. The goal of local explanation is to explain a specific prediction output, such as the prediction of individual output. On the other hand, the purpose of global explanation is not to explain a specific instance but rather to explain the behavior of the model as a whole. The key contributions of this paper can be summarized as follows:

- We developed the ML models to classify the ischemic stroke group and the healthy control group for acute stroke prediction in an active state.
- We have used Eli5 to explain the behavior of the model as a whole to determine the significant features, which contribute to stroke prediction models.
- Further, we have utilized the LIME (Local Interpretable Model-Agnostic Explanations) method to interpret the prediction done by the model locally through a plethora of contributions from distinct EEG features.

The remainder of this article is structured as follows. Section 2 discusses experimental materials and methodology. Section 3 presents the results of our research, and Section 4 concludes with a discussion. Finally, Section 5 highlights our conclusion and future work direction.

## 2. Materials and Methods

### 2.1. EEG Data Description

**Figure 1:**
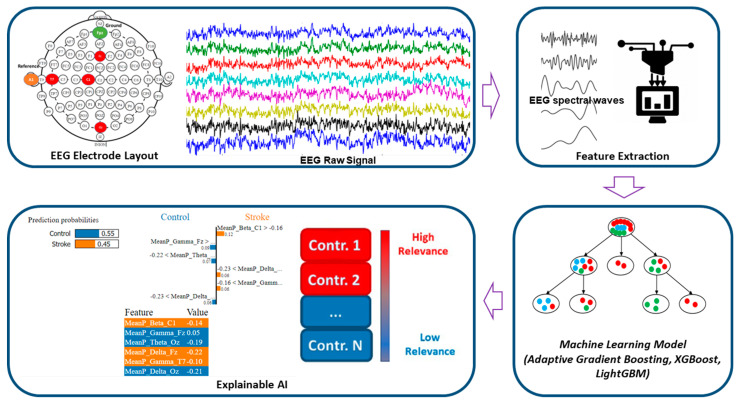
Overview of EEG-based stroke diagnostics using the explainable machine-learning approach.

The dataset included four-channel EEG recordings of stroke patients and healthy adults using the Biopac MP 160 Module (Biopac Systems Inc., Goleta, CA, USA) [5]. EEG, the electrical activity of the cerebral cortex, was constantly recorded with a wireless device at a sampling rate of 1000 Hz data. EEG data were recorded on the Fz, Oz, C1, and T7 locations using the standard 10–20 EEG system, as shown in Figure 1. The stroke group consisted of 48 stroke patients (average age: 72.2 ± 5.6 years, 62% male), and the healthy control group comprised 75 healthy adults (average age 77 years, 31% male). The frontal lobe is represented by the electrode Fz; the occipital lobe is represented by the electrode Oz; the central lobe is represented by the electrode C1, and the temporal lobe is represented by the electrode T7. Additionally, a one-channel vertical electrooculogram (VEOG) was captured to reduce eye blink artifacts and a one-channel chin electromyogram (EMG) was collected to eliminate muscle artifacts. EEG data from the stroke subjects were collected in the active state no later than three months after the diagnosis of ischemic stroke. The active state, which includes walking, working, and reading, was used in this study. In general, eye blinks and muscle artifacts impact the EEG signal during active states. We used the EOG and chin EMG to eliminate eye blink and muscle artifacts. The dataset included 48 stroke survivors and 75 healthy people. Seven stroke patients had a mild stroke (NIHSS: 1–4), ten had a moderate stroke (NIHSS: 5–15), 13 had a moderate-to-severe stroke (NIHSS: 16–20), and eighteen had a severe stroke (NIHSS: 21–42). The severity of stroke patients was measured using the National Institute of Health Stroke Scale (NIHSS) score as a guideline. The subjects in the Control group never had an ischemic stroke, a hemorrhagic stroke, or any other known neurological disease.

### 2.2. EEG Signal Pre-Processing

EEG is highly sensitive to the powerline, muscular, and cardiac artifacts and the raw EEG data accompanies those artifacts [5,21,25]. First, the original EEG data was processed to remove 60 Hz AC noise. To eliminate ocular and muscle aberrations from the waveform, independent component analysis (ICA) was used. For noise removal in the EEG data, we employed the FastICA methods [26]. EOG and EMG data were used by ICA to extract the EEG signal from the eye movements and muscle artifacts. A band-pass filter was used to filter the EEG waveform within the frequency range of 0.5–44 Hz. The EEG data were preprocessed and features were extracted with the AcqKnowledge version 5.0 (Biopac Systems Inc., Goleta, CA, USA).

### 2.3. Feature Extraction

The artifact-free EEG data was utilized to extract unique EEG frequency-specific wave patterns including delta, theta, alpha, beta, and gamma [21,27,28]. The Welch periodogram estimation approach was used to analyze EEG spectral signals in this investigation [29]. Using the Fast Fourier Transform (FFT) approach, the power spectral density (PSD) was estimated from noise-free EEG data with a 10% hamming window. The EEG signal was separated into epochs of 10 s each with a specified width of time. Mean power features were extracted within frequency ranges. A total of twenty EEG spectral power features were utilized for training the ML models.

The relative power (RP) of the EEG was estimated using FFT on the EEG signal with a 10% hamming window. We retrieved absolute power in the following spectral bandwidths: delta (δ) waveform was described between 0.5 and 4.0 Hz, theta (θ) waveform between 4.0 and 8.0 Hz, alpha (α) waveform between 8.0 and 13.0 Hz, beta (β) band between 13.0 and 30 Hz, and gamma (γ) band between 30.0 and 44 Hz. Each epoch of 10 s was used to assess all EEG power features. Equation (1) defined relative band power.

(1) $$p_{j}=\frac{P_{j}}{\sum_{j\;=1}^{q}P_{j}}$$

Here P_j_ is absolute spectral power density at frequency j (with j = 1,2, …, q) and q denotes the frequency range 0.5–4 Hz, 4–8 Hz, 8–13 Hz, and 13–30 Hz.

### 2.4. Features Scaling

Any of the features in our dataset did not contain any NaN or missing values and we implemented various feature scaling methods. Among various feature scaling techniques, normalization and standardization are two of the most widely used methods to scale the training instances before feeding them to different ML algorithms. ML algorithms like linear regression, k-means clustering, etc., which use gradient descent as an optimization method, are largely dependent on the distribution of the dataset. If the range of features is large, then normalization is important for those algorithms because gradient descent can optimize the weights better if the training instances are normalized. But in our case, we can see that the data was following a skewed distribution, and the standard scaling method works better in our case than the normalization technique. We used the Scikit-Learn library [30] to add the scaling techniques for features to our datasets.

**Figure 2:**
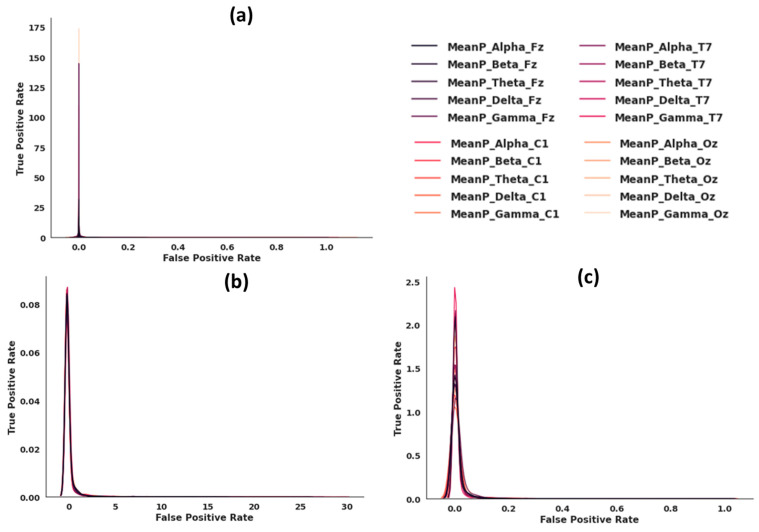
(**a**) Data distribution with no feature scaling. (**b**) Data distribution with standard feature scaling. (**c**) Data distribution with min-max feature scaling.

Figure 2 illustrates data distributions without feature scaling (a), with standard feature scaling (b), and with min-max feature scaling (c). We applied a variety of scaling strategies to get all features into the same range. We observed that the standard feature scaling method produced the best distribution, so we used this technique.

### 2.5. Machine-Learning Classification Algorithms

We investigated ML methods for the automated diagnosis of ischemic stroke patients and healthy people based on neural characteristics of active states, including walking, working, and cognitive reading activities. A training dataset was made up of 80% of the EEG feature data, and a test dataset was made up of 20%. To distinguish between the neurological features of acute patients and those of healthy individuals, the Adaptive Gradient Boosting, XGBoost, and LightGBM models were implemented.

#### 2.5.1. Adaptive Gradient Boosting (AdaBoost)

When it comes to “boosting” algorithms, we are referring to those used to lower the number of mistakes made by “weak” learners. These “weak” learners consistently provide classifiers that are just marginally better than random guessing [31]. Multiple weak learners are successively trained on the training dataset, and the weights of erroneously classified samples are adjusted so that subsequent classifiers can concentrate on more difficult instances.

This can be accomplished by minimizing the exponential loss function,

(2) $$\epsilon_{t}=\frac{1}{2}\underset{\left(i,y\right)\epsilon B}{\sum }D_{t}\left(i,y\right)\left(1-h_{t}\left(x_{i},y_{i}\right)+h_{t}\left(x_{i},y\right)\right)$$

where, $\epsilon_{t}$ denotes the loss function, $D_{t}$ is the distribution of the dataset, i is the number of training example and y is the class labels, and $h_{t}$ denotes the hypotheses function.

The exponential loss function is highly optimized, and it is very consistent with the objective function of decreasing classification error due to its elegant and easy updating formula. It is also supported by its standard log likelihood.

Finally, the classification result is obtained using additive weighted combination of the weak learners,

(3) $$H\left(x\right)=\overset{T}{\underset{t=1}{\sum }}\alpha_{t}h_{t}\left(x\right)$$

where, $h_{t}\left(x\right)$ is the output of the weak learner t for input x, and $\alpha_{t}$ is the weight assigned to a weak learner.

#### 2.5.2. XGBoost

XGBoost [32] is another ensemble machine learning technique whose goal is not to depend on a single decision tree, but rather to employ several trees to forecast the final class label. It nearly sounds like previous ensemble-based boosting algorithms up to this point, but the strength of XGBoost comes in its scalability. Its implementation enables hardware and software level optimizations like parallelization, tree pruning, cache awareness, sparsity awareness, and data compression, among others. By adding these improvements, XGboost becomes a very scalable ML method that can solve many important real-world problems with few resources.

#### 2.5.3. LightGBM

Traditional GBDTs (Gradient Boosted Decision Trees) must analyze the whole sample space for each feature to approximate the information gain of all possible partition points. As a result, the computational complexity grows in direct proportion to the number of training instances and feature sets. When dealing with a large number of data points, these GBDT implementations become computationally costly and time-consuming. Two novel strategies have been presented by LightGBM (Light Gradient Boosting Machine) [33]. The first strategy is Gradient-Based One-Side Sampling (GOSS). To calculate information gain, the GOSS method excludes the portion of data instances with lower gradients and uses just the remaining data instances. The second strategy is Exclusive Function Bundling (EFB). It attempts to minimize the high computing costs and ineffectiveness of earlier GBDT (Gradient Boosted Decision Trees) methods. In addition, the EFB approach can effectively reduce the number of features by mutually exclusive grouping characteristics without compromising the accuracy of the overall forecast.

### 2.6. Machine-Learning Analysis and Performance Matrix

We have performed the experiments on the Google Colaboratory environment. Google Colab provides us with 16 GB of RAM, and a 2-core Intel Xeon Processor for our experiments. We have used the Scikit-Learn library which provides us with a plethora of functionalities, for example, Decision Tree, Random Forest classifiers, and different methods like Standard Scaling, Min-Max Scaling etc., for classification and feature scaling tasks, respectively. We took advantage of the Eli5 and LIME library to make our “Black Box” ML models explainable by assigning weights to different features which signifies their importance in classification. We utilized the Seaborn and Matplotlib libraries for data visualization to better understand the distribution of our dataset and to generate the data visualization for our results.

From the confusion matrix of ML model, sensitivity (true positive rate), specificity (true negative rate), precision (positive predictive rate), negative predictive value, accuracy (ACC), area under the curve (AUC), and Gini coefficient were evaluated. The following are the formulae used to calculate the performance evaluation metrics:

(4) $$\mathrm{Sensitivity}=\frac{\mathrm{TP}}{\mathrm{TP}+\mathrm{FN}}$$

(5) $$\mathrm{Specificity}=\frac{\mathrm{TN}}{\mathrm{TN}+\mathrm{FP}}$$

(6) $$\mathrm{Precision}=\frac{\mathrm{TP}}{\mathrm{TP}+\mathrm{FP}}$$

(7) $$\mathrm{Negative}\;\mathrm{predictive}\;\mathrm{value}\;\left(\mathrm{NPV}\right)=\frac{\mathrm{TN}}{\mathrm{TN}+\mathrm{FN}}$$

(8) $$\mathrm{Accuracy}\left(\mathrm{ACC}\right)=\frac{\mathrm{TN}+\mathrm{TP}}{\mathrm{TN}+\mathrm{TP}+\mathrm{FN}+\mathrm{FP}}$$

where TP denotes the true positive, TN stands the true negative, FP represents the false positive, and FN denotes the false negative.

### 2.7. Explainable Artificial Intelligence (XAI)

#### 2.7.1. Eli5

Eli5 [34] is a Python module that converts “black box” machine learning models to “white box” models by explaining the model. It is compatible with several ML libraries, including the Scikit-Learn library, Keras, and XGBoost models, among others. It is not a model-independent library since it implements the XGBoost model. The Eli5 technique closely resembles Random Forest feature weight evaluation. The weights are calculated by following the decision splits in the ensemble of trees. The contribution score is calculated by the degree to which the output score of each node varies from parent to child node. The prediction is based on the sum of the feature contributions and the “bias” score. By including permutation significance, ELi5 directs its attention to the selection of features. Extraction and presentation of feature weights and their contribution from the model as a form of global explanation are made possible using this library. The tabular presentation of the characteristics and their weights serves as the foundation for the visualizations. There is no way to change the model’s judgments, and the features that influence the model’s choices are ordered by their importance.

#### 2.7.2. LIME

Local Interpretable Model Agnostic Explanations, LIME [35] is a method that can make an ML model understandable while remaining model agnostic. LIME defines the model explanation by the following formula:

(9) $$\xi \left(x\right)=\arg\underset{g\in G}{\min}ℒ\left(f,g,\pi_{x}\right)+\Omega \left(g\right)$$

where G is denoted as a set of interpretable models, and the (g) denotes the complexity of the explanation g∈G. The objective is to reduce (g) so that the simpler models can also be interpreted. The $ℒ\left(f,g,\pi_{x}\right)$ represents a measure of how closely the explanation model g matches of the original model’s prediction, also known as fidelity. Local fidelity refers to the need for the explanation to accurately represent the classifier’s behavior “around” the instance being predicted without peeking into the model, thus the model-agnostic approach.

The LIME model generates outputs in visual figures that are divided into three sections: the feature probabilities on the left, the feature probabilities on the right, and the feature value table on the bottom side of the figure. The predicted values of the probabilities are located on the left. The graph of prediction probabilities illustrates the model’s judgment on an instance of the test dataset, i.e., the result it predicts and the probability that corresponds to that outcome.

## 3. Results

### 3.1. Stroke Prediction Model Using Machine Learning Approach

**Figure 3:**
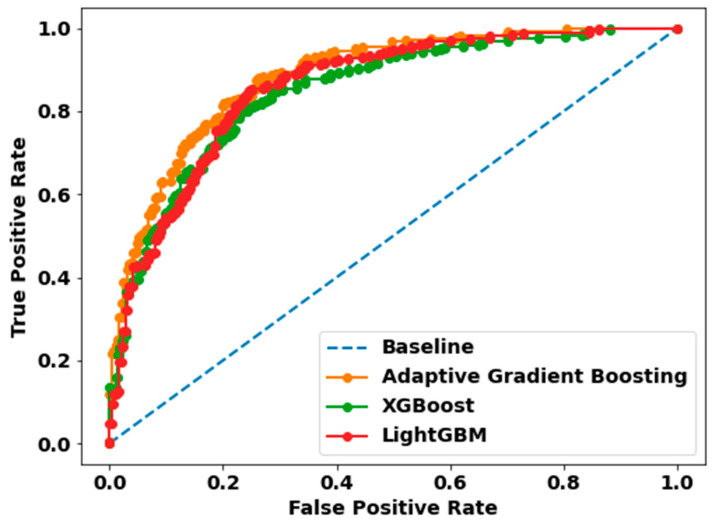
Receiver Operating Characteristic (ROC) curves of the Adaptive Gradient Boosting, XGBoost, and LightGBM classification models. The orange ROC curve represents Adap-tive Gradient Boosting with AUC (80%), the green ROC curve denotes XGBoost with AUC (77%), and the red ROC curve refers to LightGBM with AUC (78%). The area under the ROC curve (AUC) indicates the accuracy of a prediction. The diagonal blue dot line is the reference line.

We experimented with several ML algorithms such as Adaptive Gradient Boosting, XGboost, and LightGBM to classify whether a person lies within a control group or is a stroke patient. We only experimented with a subset of data that represents healthy adults that are in an active state, i.e., they are either working, reading, or walking. ROC represents the Receiver Operator Characteristics are a statistic that defines a classification’s validity. Figure 3 depicts a ROC curve for each model, with each graph including two curves: the ROC curve, which represents a prediction, and the baseline, which is a threshold that signifies a minimal or beginning point in use for comparisons.

#### 3.1.1. Hyperparameter Tuning

To get the best parameters that would give us the best results, we used the “Grid SearchCV” method provided by the “Scikit-learn” library. Table 1 includes the tuned hyperparameters for different ML models.

#### 3.1.2. ML Classification Results

The overall classification performance of the stroke prediction models are illustrated in Table 2. This performance table consists of precision (positive predictive rate), recall, F1 score, accuracy, and AUC, which are all generated from the confusion matrix. These components are then reassembled to form the complete scenario of the performance of the various ML algorithms in the aforementioned classification task. Figure 3 exhibits ROC curves, which illustrate the performance curves of classification models using test datasets. The evaluation of these models is based on the ROC curves. According to Table 2, it is fairly evident that the Adaptive Gradient Boosting algorithm outperforms all other methods across all criteria. This is true regardless of the methodologies being compared. For classifying the Stroke Group, the Adaptive Gradient Boosting algorithm achieved 82% precision, 78% recall, 80% F1 score, 80% accuracy, and 80% AUC, whereas the LightGBM method achieved 80% precision, 76% recall, 78% F1 score, 78% accuracy, and 78% AUC. In addition, the other boosting algorithms, such as XGBoost achieved comparable results in terms of evaluation metrics mentioned above with only a 2–3% performance degradation in each metric. In conclusion, Adaptive Gradient Boosting is the most effective algorithms for classifying a Stroke patient based on active-state EEG data. In spite of this, the other boosting techniques performed competitively in all performance metrics. However, it is not clear which of the features such as, the different bands of the EEG signals, i.e., gamma, delta, theta etc., contributed significantly to correctly classify a stroke patient which in turn reflected in the overall performance of the ML algorithms.

### 3.2. Explanations of ML Models

We utilized XAI tools such as Eli5 and LIME to determine how a model predicts and to explain how attributes contributed to the prediction. Because we are distinguishing between the Control and Stroke groups, it is vital to examine how our models allocate each data instance to the right category based on the features we supply. If we can identify the main traits that significantly contribute to the prediction, we will be able to reduce the feature space, which will save time during model training and increase its accuracy. In addition, we will be able to focus our efforts on acquiring additional data points that include just those qualities, thus improving the accuracy of model prediction.

#### 3.2.1. Model Explanation using Eli5

**Figure 4:**
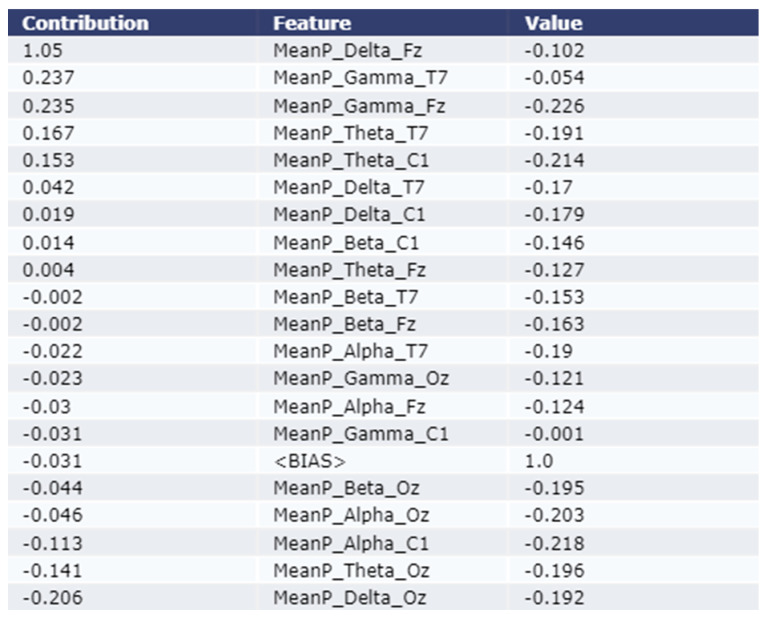
Explanation of the local contribution of EEG features through the Eli5 model in classifying a single test instance (predicted class = Stroke) using the XGBoost model. The importance of the features is denoted by positive contribution.

**Figure 5:**
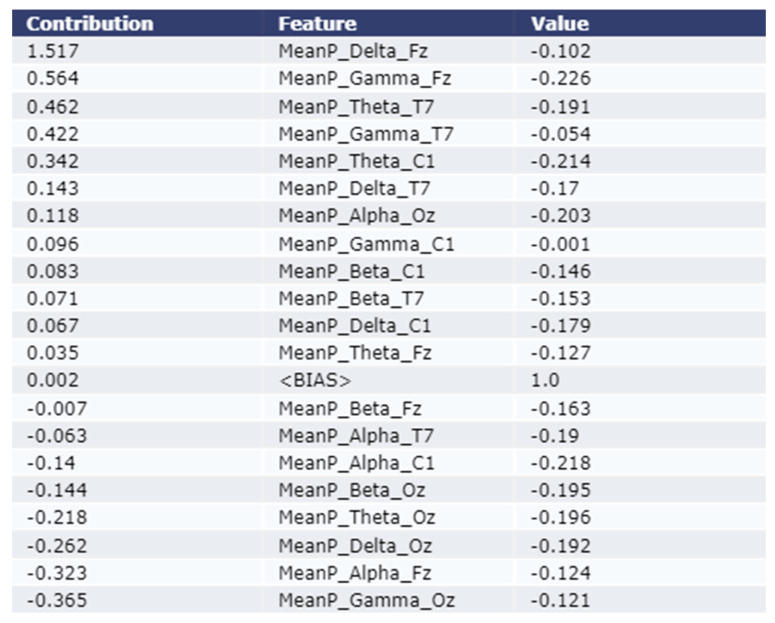
Explanation of the local contribution of EEG features through the Eli5 model in classifying a single test instance (predicted class = Stroke) using the LightGBM model. The importance of the features is denoted by positive contribution.

As shown in the Figure 4 and Figure 5, the Eli5 model is used to get the major feature local contribution towards predicting a test sample as Stroke class by employing XGBoost Model and LightGBM model respectively. Eli5 model analysis was not performed for the Adaptive Gradient Boosting model due to lack of Eli5 ADABoost library. The importance is denoted in the contribution column of the table. The test instance was passed to the trained ML model which predicted the instance as class 1 (Stroke Group). The Eli5 methodology suggests that the ML models assigned frontal delta, frontal gamma, and central theta waves higher weight than other features. This trend remains consistent for all five classification models employed in this study.

As shown in Figure 4, the most contributing feature are MeanP_Delta_Fz, MeanP_Gamma_T7, and MeanP_Gamma_Fz for an example test instance using the XGBoost model. The contribution of these three features are 1.05, 0.237, and 0.235 respectively. As shown in Figure 5, the most contributing feature are MeanP_Delta_Fz, MeanP_Gama_Fz, and MeanP_Theta_T7 for an example test instance using the LightGBM model. The contribution of these three features are 1.517, 0.564, and 0.462 respectively.

#### 3.2.2. Model Explanation using LIME

**Figure 6:**
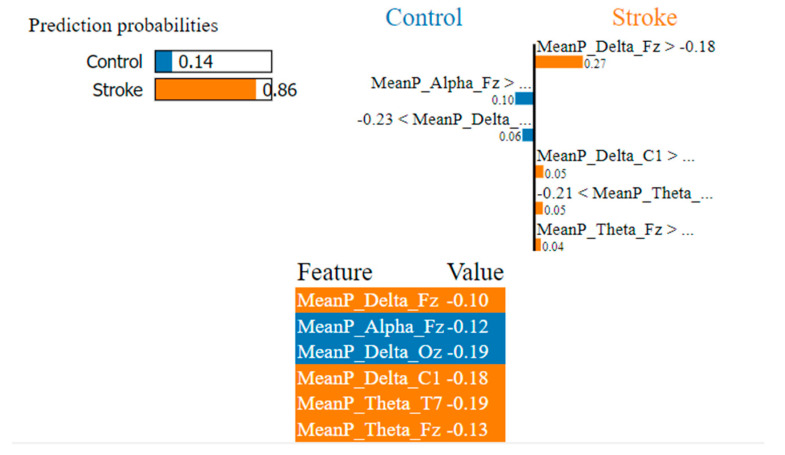
Visualization of the local contribution of EEG features through the LIME model in classifying a single test instance (predicted class = Stroke) using the Adaptive Gradient Boosting model. The orange-marked cells represent the features that contributed most to classifying the Stroke class.

**Figure 7:**
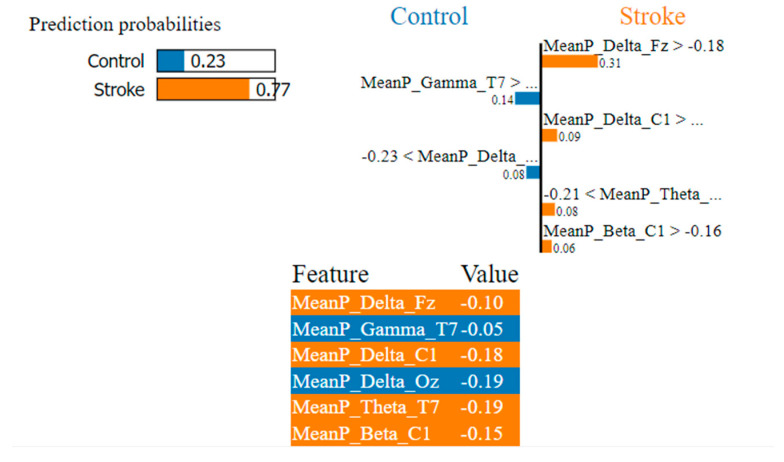
Visualization of the local contribution of EEG features through the LIME model in classifying a single test instance (predicted class = Stroke) using the XGBoost model. The orange-marked cells represent the features that contributed most to classifying the Stroke class.

**Figure 8:**
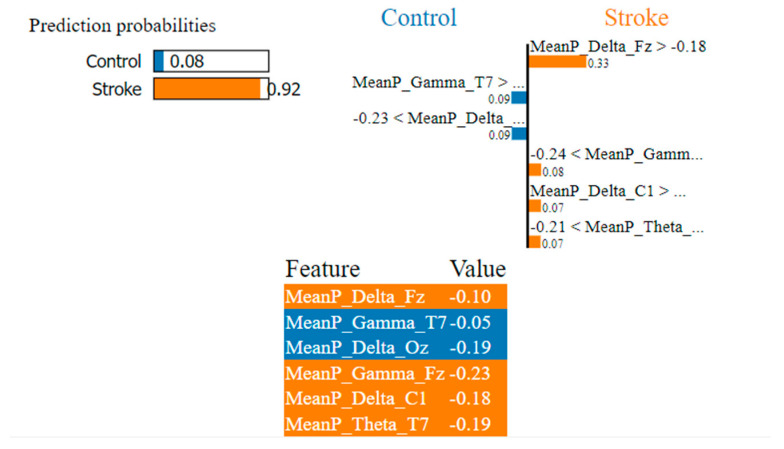
Visualization of the local contribution of EEG features through the LIME model in classifying a single test instance (predicted class = Stroke) using the LightGBM model. The orange-marked cells represent the features that contributed most to classifying the Stroke class.

As shown in Figure 6, Figure 7 and Figure 8, the LIME model is applied to the data for determining how a model predicts and to explain how attributes contributed to the prediction of an instance, stroke class, and control class. The important features are consistent across all models.

In Figure 6, the LIME visualization is reported for the Adaptive Gradient Boosting model to predict an instance, the control or stroke class. The predicted probability of the test stroke instance and control instance is 86% and 14% respectively. The three most contributing features of this model are MeanP_Delta_Fz, MeanP_Delta_C1, and MeanP_Theta_T7 to predict a stroke instance. The feature importance of these three features are 27%, 5%, and 5% respectively. Higher frontal and central delta and theta have a greater contribution to the prediction of stroke using the AdaBoost model.

In Figure 7, the LIME visualization is reported for the XGBoost model to predict an instance, the control or stroke class. The predicted probability of the test stroke instance and control instance is 77% and 23% respectively. The three most contributing features of this model are MeanP_Delta_Fz, MeanP_Delta_C1, and MeanP_Theta_T7 to predict a stroke instance. The feature importance of these three features are 31%, 9%, and 8% respectively. Higher frontal and central delta and temporal theta have a much greater contribution to the prediction of stroke using the XGBoost model.

In Figure 8, the LIME visualization is reported for the LightGBM model to predict an instance, the control or stroke class. The predicted probability of the test stroke instance and control instance is 92% and 8% respectively. The three most contributing features of this model are MeanP_Delta_Fz, MeanP_Gamma_Fz, and MeanP_Delta_C1 to predict a stroke instance. The feature importance of these three features are 33%, 8%, and 7% respectively. Higher frontal delta and gamma, central delta have a much greater contribution to the prediction of stroke using the LightGBM model.

## 4. Discussion

In this work, we aimed to interpret the stroke prediction ML model locally using EEG data from stroke patients and healthy people during an active state (walking, working, and reading states). Specific EEG band power is associated with the specific functional outcome of the brain and, in the case of ischemic stroke, is linked to the degree of neural impairment in the lesion area of the brain.

This work employs three state-of-the-art machine learning models to classify stroke patients in their active state: Adaptive Gradient Boosting, XGBoost, and LightGBM. Although the XGBoost model and LightGBM fared very well in terms of classification metrics (such as precision, recall, F1 score, accuracy, AUC, etc.), the Adaptive Gradient Boosting technique outperformed the other models in every criterion. In the active stage, the precision and recall scores of the Adaptive Gradient Boosting model are significantly higher than those of the other ML models, indicating that it is the best-suited model for distinguishing between a stroke patient and a non-stroke patient with more accuracy and reliability.

According to the findings of our ML explanation, the Eli5 interpretable model described frontal and central delta, theta, and, gamma waves with higher weight for classifying stroke patients in ML models. Our results demonstrated that the impact of these features is substantial for all the classification models that were used in this study. For stroke patient classification, the LIME model prioritized the frontal and central delta and theta features. Both the Eli5 and LIME interpretable models give the most weight to the delta, and theta waves when classifying stroke patients. This trend has persisted across all classification methods used in this research and is supported by our previous studies [5,21].

The slow-wave delta activity is considered the most reliable prediction measure compared with faster wave activity. Delta activity is believed to derive from nerve cells in the thalamus and deep cortical layers. Delta wave may indicate hyperpolarization and curbing of cortical nerve cells, hindering neural activity. Abnormal delta activity is often associated with brain injury (lesion) location [36]. Several studies revealed higher delta power in the electrodes of the cortical positions in post-stroke EEG [5,6]. The delta-alpha ratio is found to be statistically significantly different between the stroke group and the healthy group [5,21].

Weaker theta activity was observed to exist in the stroke class relative to that of the control class in active states [5]. Although theta was found as an unreliable measure to predict post-stroke pathology, functional outcome, and cognitive impairment [21,37], few studies revealed theta activity as a prospective biomarker of post-stroke pathology and capable of discriminating between stroke group and healthy adults [38], predicting functional outcomes [39], and cognitive deficits [40].

In this study, we conducted EEG only with four channels for exploring changes in EEG for neural impairment due to ischemic stroke. Although those cortical positions resemble other cortical positions, there is still the chance of missing lesion locations. So, the outcomes of the statistical investigation were limited to a few cortical electrodes. In the future, we will extend our study with multimodal biosignal data for automated stroke prognosis and post-stroke rehabilitation studies.

## 5. Conclusions

We investigated explainable machine-learning methods for automated diagnosis of ischemic stroke patients and healthy people using neural features of active states such as walking, working, and cognitive reading. ML algorithms were also used to classify stroke patients and healthy people. When identifying stroke patients, both the Eli5 and LIME interpretable models place the greatest importance on the delta and theta waves. It is expected that the results of this research into explainable artificial intelligence will help with treating and rehabilitating people who have had a stroke, as well as making it easier for doctors to explain their diagnoses.

## Figures and Tables

**Table 1:** Hyperparameters for training machine learning models.

ML Model Hyperparameters	AdaBoost	XGBoost	LightGBM
max_features	--	--	--
max_depth	--	3	--
criterion	--	--	--
learning_rate	0.1	0.1	0.1
n_estimators	300	300	300
base_estimator__max _depth	8	--	--
base_estimator__min _samples_leaf	10	--	--

**Table 2:** The Classification Performance of Various Machine Learning Models for the Stroke Group classification using EEG data.

Model	Precision	Recall	F1-Score	Accuracy	AUC
Adaptive Gradient Boosting	0.82	0.78	0.80	0.80	0.80
XGBoost	0.79	0.74	0.77	0.77	0.77
LightGBM	0.80	0.76	0.78	0.78	0.78

## Data Availability

Not applicable.
